# *Staphylococcus aureus*-associated infected subclavian artery pseudoaneurysm treated with dual bare-metal stent implantation: a case report

**DOI:** 10.3389/fcvm.2026.1965965

**Published:** 2026-10-22

**Authors:** Ruihua Li, Shuohao Shen, Yuanbin Cao, Jianjun Jiang, Xiangjiu Ding

**Affiliations:** 1 Department of Vascular Surgery, General Surgery, Qilu Hospital (Qingdao), Cheeloo College of Medicine, Shandong University, Qingdao, China; 2 Department of Vascular Surgery, General Surgery, Qilu Hospital of Shandong University, Jinan, China; 3 Shandong Key Laboratory of Medicine and Prevention Integration in Rheumatism and Immunity Disease, Qilu Hospital of Shandong University, Jinan, China

**Keywords:** bare-metal stent, endovascular repair, infected aneurysm, *Staphylococcus aureus*, subclavian artery aneurysm

## Abstract

Infected subclavian artery aneurysms are exceedingly rare, life-threatening vascular lesions associated with high risks of rupture and mortality. We report a 70-year-old male presenting with intermittent hemoptysis, who was diagnosed with a pseudoaneurysm of the left subclavian artery adjacent to the dominant vertebral artery. Blood cultures confirmed methicillin-sensitive *Staphylococcus aureus* infection. The patient underwent endovascular repair with dual bare-metal stents to preserve vertebral artery perfusion. Hemoptysis completely resolved post-intervention. Complete thrombosis and occlusion of the aneurysm sac were achieved at the 3-month follow-up, with remarkable sac shrinkage observed at 6 months. This case suggests that dual bare-metal stents may represent a feasible potential treatment alternative for highly selected patients with infected subclavian artery aneurysms. Long-term safety and efficacy remain to be further verified. Long-term antimicrobial therapy and close surveillance remain mandatory.

## 1 Introduction

Subclavian artery aneurysms (SAAs) are rare peripheral vascular lesions, accounting for approximately 1% of all peripheral arterial aneurysms (1, 2). Their etiologies are diverse, including atherosclerosis, chronic vascular compression secondary to thoracic outlet syndrome, connective tissue disorders, trauma, iatrogenic damage, and infection (2–4). Infected subclavian artery aneurysms (ISAAs) represent an exceptionally rare but highly fatal subtype, resulting from microbial invasion and inflammation destruction of the arterial wall (5, 6). These infected aneurysms confer a high risk of rupture and mortality, leading to generally poor clinical prognoses (7, 8).

The clinical management of ISAAs is challenging (9, 10), and current therapeutic options consist of open surgical repair (OSR), hybrid surgery, and endovascular intervention. OSR has traditionally been considered the gold standard. However, it is associated with significant trauma, prolonged hospitalization, and a high incidence of postoperative complications, such as recurrent graft infection, local abscess formation, and persistent bacteremia (11, 12). Hybrid surgery, combining endovascular exclusion with open debridement or bypass reconstruction, has been sporadically adopted for complex ISAAs (6). Nevertheless, these procedures still require invasive dissection and carry an inherent risk of infection.

In recent years, endovascular techniques have been widely adopted for peripheral artery aneurysms repair due to their minimal invasiveness. However, unlike true aneurysms, infectious arterial lesions theoretically require thorough debridement of infected tissue and drainage of purulent foci, rendering the endovascular approach for ISAAs particularly controversial (13). To date, published reports focusing on endovascular treatment for ISAAs remain limited (13–16), and no standardized therapeutic protocol has been established.

Biofilm formation by *Staphylococcus aureus* embedded within the aneurysm wall renders antibiotic monotherapy ineffective (17); meanwhile, stent-grafts further prevent systemic antibiotics from infiltrating into infectious lesions. Herein, we report an individualized endovascular strategy for *S. aureus-*associated ISAA to resolve this therapeutic dilemma. To our knowledge, this constitutes the first clinical report describing dual bare-metal stent (BMS) implantation for symptomatic infectious subclavian artery aneurysm, providing a minimally invasive option for selected patients.

## 2 Case report

A 70-year-old male patient presented with intermittent hemoptysis, without fever, chest pain, or dyspnea. The patient had a 15-year history of femoral head replacement and underwent revision hip arthroplasty 3 months prior to admission. Two months before presentation, he developed left femoropopliteal venous thrombosis, for which he underwent percutaneous mechanical thrombectomy and temporary inferior vena cava filter implantation; the filter was removed 1 month later. He was treated with rivaroxaban at a dose of 15 mg twice daily for 3 weeks, then switched to 20 mg once daily thereafter. He had no history of pulmonary embolism, bronchiectasis, or pulmonary malignancy. He denied intravenous drug use and any other prior procedures. Physical examination revealed no lower-limb edema, and the hip surgical incision was well-healed, without local erythema, swelling, or purulent discharge.

Laboratory tests showed normal white blood cell count and procalcitonin. Notably, the erythrocyte sedimentation rate (ESR, 150 mm/h) and high-sensitivity C-reactive protein level (hs-CRP, 44.8 mg/L) were significantly elevated, while the D-dimer level was mildly increased (1.9 μg/mL). Tumor markers, routine coagulation tests, and rheumatological screening findings were all within normal reference ranges.

Initial non-contrast chest computed tomography (CT) revealed an irregular 38 mm × 16 mm soft-tissue mass in the left upper lobe, suspicious for malignancy. Subsequent contrast-enhanced CT further identified a 30-mm pseudoaneurysm of the left subclavian artery with severe proximal stenosis of the dominant left vertebral artery (Figures 1A,B). The proximal subclavian artery diameter was approximately 13 mm. Pulmonary embolism and malignancy were excluded. Blood cultures grew methicillin-sensitive *Staphylococcus aureus* (MSSA). Transthoracic echocardiography showed no valvular vegetations, excluding infective endocarditis. Venous ultrasonography revealed minimal residual popliteal venous thrombus. Hip ultrasonography revealed no peri-prosthetic or adjacent soft-tissue abnormalities. A diagnosis of infected left subclavian artery pseudoaneurysm was established. The hemoptysis was presumed to result from erosion of the pseudoaneurysm into the adjacent bronchial wall.

**Figure 1:**
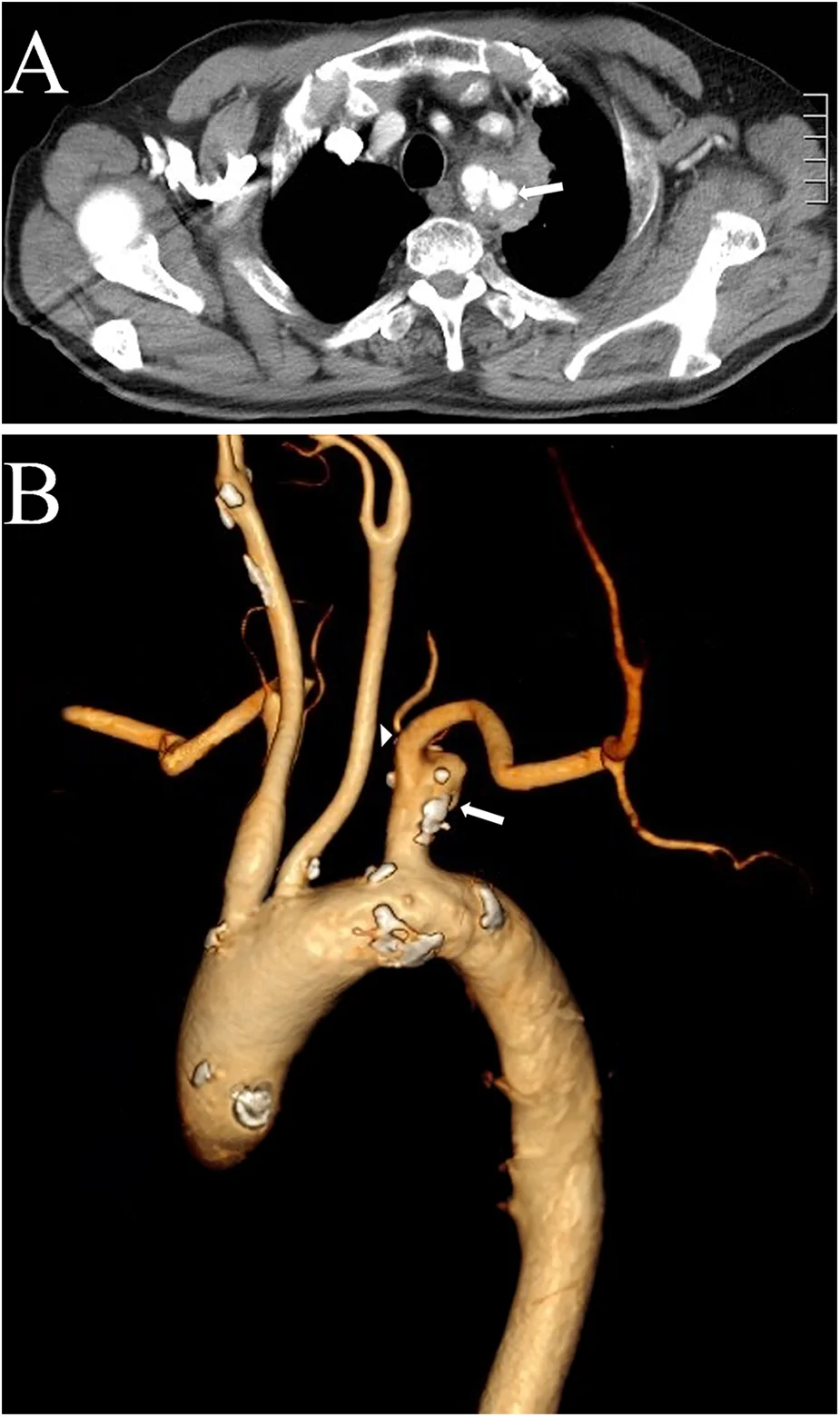
Preoperative contrast-enhanced computed tomography (CT). **(A)** Axial scan showed a 30-mm pseudoaneurysm (white arrow) of the left subclavian artery compressing the adjacent pulmonary parenchyma. **(B)** CT image with three-dimensional reconstruction demonstrated the subclavian artery pseudoaneurysm (white arrow) with proximal stenosis (white triangle) of the dominant left vertebral artery.

Rivaroxaban was discontinued upon admission, but hemoptysis persisted. Empirical intravenous ceftriaxone (2 g once daily) and vancomycin (1 g every 12 h, dose adjusted based on renal function and therapeutic drug monitoring) were initiated. After confirmation of MSSA, the regimen was switched to targeted therapy with intravenous cefazolin (2 g every 8 h). Given the rupture risk of the aneurysm, surgical intervention was indicated. Clinically, the patient was technically a candidate for OSR, which was the first-line recommended treatment. However, after full informed consent discussion regarding the substantial perioperative risks, the patient and family declined OSR. Therefore, endovascular repair was selected as an alternative treatment option.

The procedure was performed under local anesthesia via the left radial artery, and a 7-French sheath was inserted. Under fluoroscopic guidance, a 5-French pigtail catheter was advanced into the ascending aorta. Diagnostic angiography confirmed a 30-mm left subclavian artery pseudoaneurysm and severe proximal vertebral artery stenosis with antegrade perfusion (Figure 2A). After guidewire exchange, two 14 mm × 60 mm BMS (Smart Control, Cordis, USA) were deployed and overlapped to cover the pseudoaneurysm orifice. Completion angiography confirmed patent stents with persistent contrast opacification within the aneurysm sac, while antegrade vertebral flow was well-preserved (Figure 2B).

**Figure 2:**
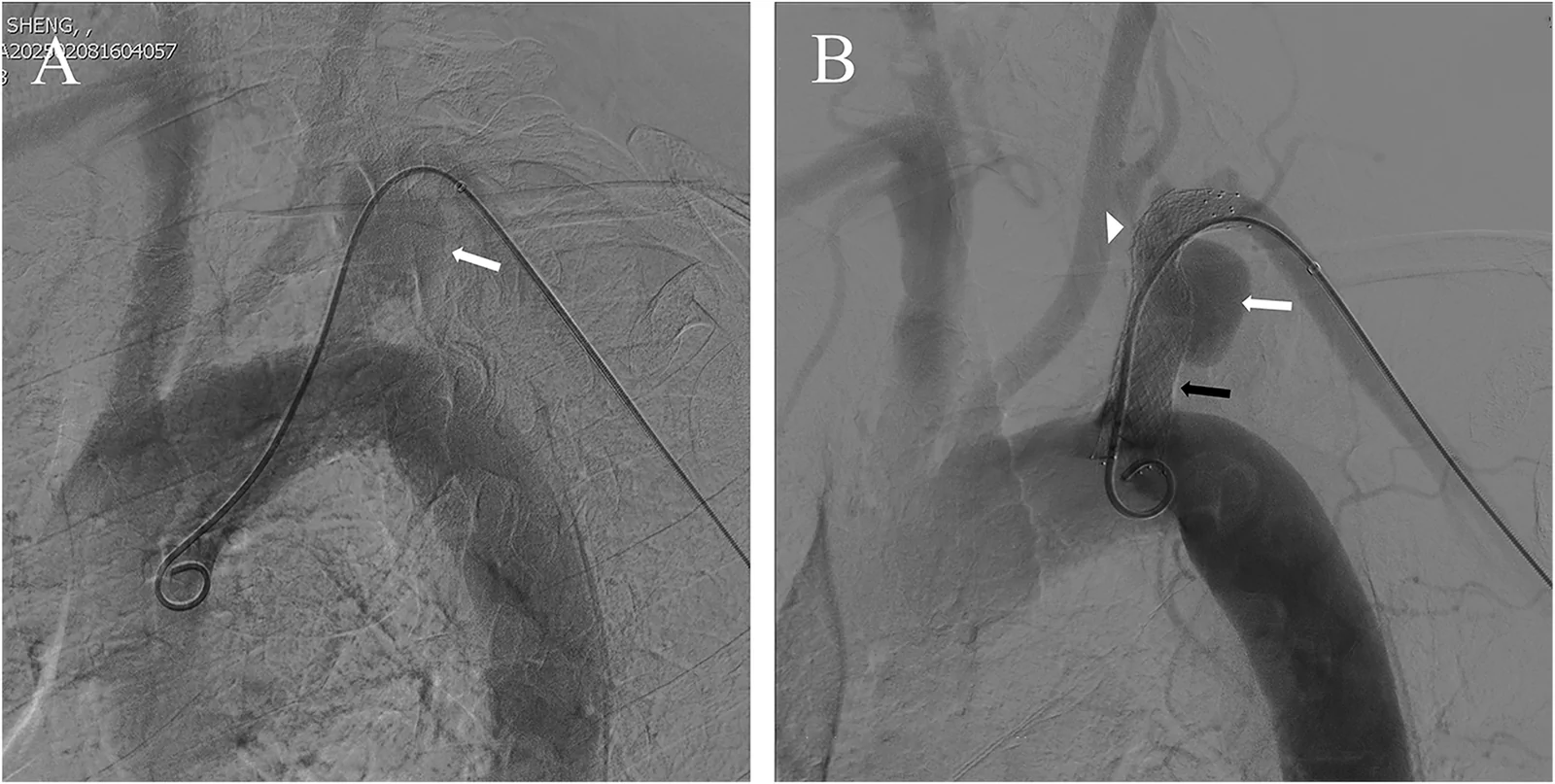
Intraoperative angiography. **(A)** Aortography confirmed the left subclavian artery pseudoaneurysm (white arrow). **(B)** Completion angiography revealed patent dual bare-metal stents (black arrow), persistent contrast opacification inside the aneurysm sac (white arrow), and well-preserved antegrade vertebral perfusion with proximal stenosis (white triangle).

The patient recovered uneventfully, with complete resolution of hemoptysis. Systemic anticoagulation was withheld postoperatively, given the hemorrhagic risk, and to facilitate sac thrombosis. Intravenous cefazolin was administered for 4 weeks, followed by oral cefuroxime axetil 500 mg twice daily for suppressive therapy. After initiation of targeted cefazolin, subsequent serial blood cultures were persistently negative. Serial inflammatory markers showed progressive normalization: ESR decreased from 150 mm/h at admission to 86 mm/h at 1 month and 12.1 mm/h at 3 months; hs-CRP decreased from 44.8 mg/L at admission to 23.9 mg/L at 1 month and 7.8 mg/L at 3 months. At the 3-month follow-up, the patient was asymptomatic. Lower-extremity venous ultrasonography revealed no evidence of extension of thrombosis. CT angiography confirmed complete thrombosis and occlusion of the aneurysm sac, with preserved patency of the subclavian and vertebral arteries. At the 6-month follow-up, CT angiography (Supplementary Movie S1) demonstrated that the maximal diameter of the aneurysm sac decreased to 20 mm, while branch vessel patency remained stable (Figures 3A,B). All inflammatory markers remained within normal ranges. At the 12-month follow-up, the patient remained asymptomatic. All inflammatory markers were negative. Antibiotics were discontinued after completion of the full 12-month course. Throughout the entire follow-up period, the patient experienced no posterior-circulation neurological symptoms, transient ischemic attack, or stroke. Long-term surveillance is currently underway. Figure 4 illustrates the patient's diagnostic and therapeutic process.

**Figure 3:**
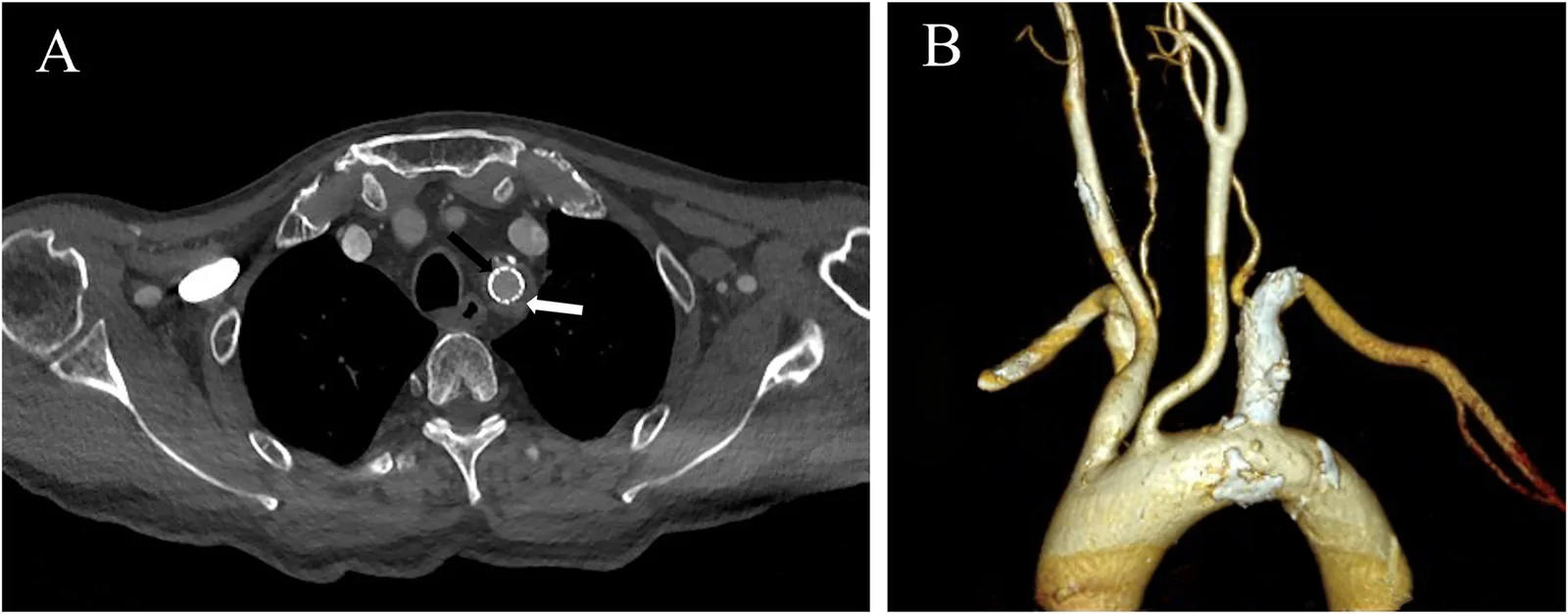
Six-month follow-up CT angiography. **(A)** Axial scan demonstrated patent stents, with reduction of the aneurysm (white arrow) to 20 mm. **(B)** CT image with three-dimensional reconstruction confirmed obliteration of the aneurysm sac and preserved patency of the left subclavian artery and left vertebral artery.

**Figure 4:**
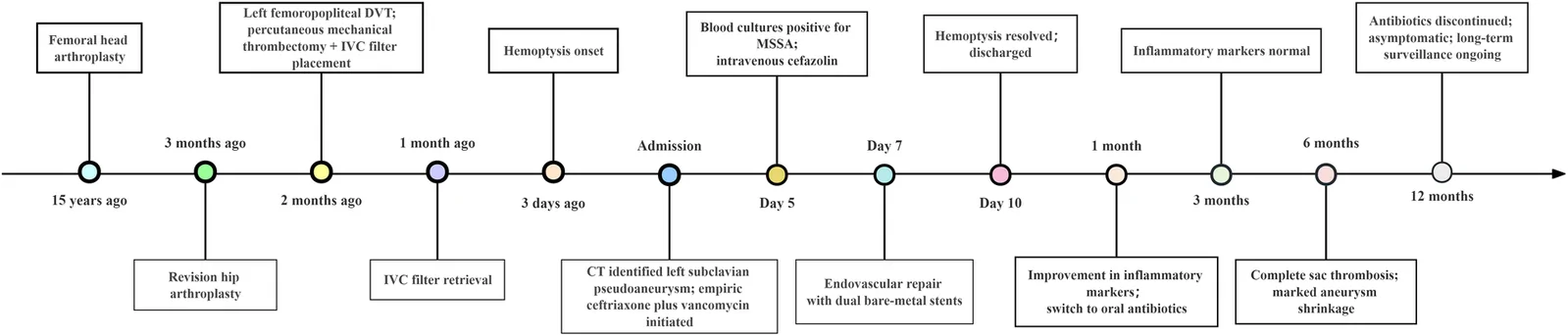
Timeline of the patient's diagnostic and therapeutic process. DVT, deep venous thrombosis; IVC, inferior vena cava; CT, computed tomography; MSSA, methicillin-sensitive *Staphylococcus aureus.*

## 3 Discussion

ISAAs are extremely rare but life-threatening. They carry catastrophic risks, including rupture, uncontrolled sepsis, and massive hemorrhage (1). The subclavian region lies in close proximity to the brachial plexus, lung apex, thoracic duct and vertebral artery. In addition, most patients have multiple underlying comorbidities (16). These factors render ISAA management technically demanding and clinically challenging.

Clinical evidence regarding ISAAs remains scarce, consisting predominantly of case reports. A 2023 scoping review including 33 patients treated from 2000 to 2023 demonstrated the dismal prognosis associated with this disease (2). Three patients (9.1%) died preoperatively due to aneurysm rupture. Of the remaining 29 patients who received definitive treatment, only 42.4% achieved complete disease resolution. Postoperative complications occurred in 27.3% of patients, and reintervention was necessary in 18.2%, with an overall mortality rate of 27.3%. In the absence of high-quality clinical evidence, contemporary therapeutic strategies remain largely individualized.

Regarding the source of infection, no definite primary infectious focus was identified in this patient. Several potential infectious foci were systematically evaluated. The patient had a history of revision hip arthroplasty and percutaneous thrombectomy combined with inferior vena cava filter placement. These invasive procedures rendered hematogenous bacterial seeding a plausible source of infection. However, no hip-related symptoms were identified, and the hip surgical incision was well-healed. Hip ultrasonography showed no peri-prosthetic fluid collection or soft-tissue lesions, essentially excluding active periprosthetic infection. Other potential sources, including skin and soft-tissue infection, urinary tract infection, and respiratory tract infection, were not identified during the diagnostic work-up. Transthoracic echocardiography also revealed no valvular vegetations, which effectively ruled out infective endocarditis as a potential etiology. Consequently, transient bacteremia originating from percutaneous endovascular intervention represents the most likely mechanism, leading to hematogenous seeding and development of the infected pseudoaneurysm.

Antimicrobial therapy and surgical intervention constitute the two cornerstones for managing ISAAs. OSR eradicates infection through aggressive debridement of infected tissue, aneurysm resection, and extra-anatomic bypass grafting (11, 18). Nevertheless, this approach is highly invasive, accompanied by high postoperative complication rates and prolonged rehabilitation, which limits its application in elderly, critically ill patients with multiple comorbidities (6, 11). By contrast, endovascular therapy features minimal invasiveness and rapid postoperative recovery, making it a viable alternative for high-risk patient cohorts (1, 2). However, implanting endovascular stent grafts inside an infected aneurysm sac increases the risk of persistent biofilm infection. Consequently, endovascular repair was adopted as an alternative to who decline OSR. Long-term suppressive antibiotics and rigorous postoperative surveillance are mandatory after endovascular repair (6, 14).

The choice of dual BMS, instead of stent-grafts or a single BMS, relied on integrated evaluation of anatomical, infectious and hemodynamic factors, plus individualized risk–benefit assessment. Anatomically, the pseudoaneurysm was adjacent to the dominant vertebral artery ostium; stent-graft implantation would occlude this orifice and raise the risk of posterior circulation stroke, whereas overlapping BMS cover the diseased parent vessel while preserving vertebral antegrade perfusion. From an infectious perspective, stent-grafts fully exclude the sac but sequester the infected lesion from systemic circulation, impairing antibiotic delivery and predisposing to persistent infection (13). Dual BMS maintain partial sac perfusion to enable antibiotic penetration. Hemodynamically, dual BMS attenuate sac inflow and lower peak intra-aneurysmal pressure. This sac had no outflow branches, and the resultant turbulence combined with reduced inflow promotes gradual sac thrombosis. Clinically, the patient had hemoptysis suggestive of a contained ruptured pseudoaneurysm. Preoperative CT showed no active free extravasation, with the sac encased by perivascular soft tissue. Although contained rupture carries high baseline rupture risk, the patient's stable clinical status supported our decision to adopt controlled low-volume sac perfusion under close surveillance. We recognize the theoretical rupture risk before complete thrombosis; therefore, intensive perioperative monitoring and serial cross-sectional imaging are essential.

Notably, severe stenosis was observed at the vertebral artery origin. Given the lack of neurological symptoms, the anatomical adjacency between the stenosis and the aneurysm, and the high bleeding risk associated with dual antiplatelet therapy, we did not perform concurrent intervention for vertebral artery stenosis.

The antithrombotic management for infected aneurysm remains challenging due to the lack of standardized relevant literature, as such individualized treatment regimens are rarely reported in clinical practice. Anticoagulation or antiplatelet therapy is inappropriate for this patient given several key clinical reasons. First, the patient presented with persistent hemoptysis, and antithrombotic treatment would increase the risk of hemorrhage. Second, antithrombotic treatment suppresses intrasac thrombosis and hinders the stabilization of infected aneurysm lesions. Third, the patient received a 14 mm BMS in the 13 mm large-caliber subclavian artery, and large-vessel stenting confers a low risk of in-stent thrombosis. Moreover, the patient had a prior femoropopliteal venous thrombosis that was adequately treated with mechanical thrombectomy and 2 months of rivaroxaban, with only minimal residual chronic thrombus detected on admission ultrasound, indicating a negligible short-term thrombotic risk. By comprehensively balancing the patient's dominant bleeding risk and extremely low thrombotic risk, we discontinued all antithrombotic agents and adopted intensive clinical and imaging surveillance, representing a reasonable individualized risk-stratified management strategy for this rare clinical scenario.

Unlike open surgical debridement, endovascular BMS implantation cannot radically resect infected arterial tissue, inevitably leaving infectious foci inside the aneurysm sac. MSAA forms mature biofilm within the aneurysm wall, which confers tolerance to regular antibiotics and easily causes recurrent infection. For all types of infected aortic/peripheral aneurysms, no unified consensus has been established on the optimal duration of antimicrobial treatment. Current clinical guidelines generally recommend a treatment course of more than 6 months, and lifelong suppressive antibiotics are required for patients with recurrent infection or persistent inflammatory lesions (19, 20). On this basis, plus the fact that this ISAA served as a metastatic septic focus of MSSA complicated bacteremia (which met diagnostic criteria for prolonged anti-infective therapy) (21), we formulated a staged 12-month antimicrobial regimen: 4 weeks of intravenous cefazolin, followed by oral cephalosporin suppressive maintenance. The decision to discontinue suppressive antibiotics at 12 months was based on comprehensive clinical, microbiological, laboratory and imaging criteria demonstrating sustained infection control. This staged antibiotic regimen combined with partial sac perfusion via dual BMS provides a novel endovascular therapeutic paradigm for MSSA-associated infected aneurysms.

Despite favorable 12-month outcomes, this study has several limitations. First, as a single-case report, its generalizability is limited, and these short-term findings do not confirm the general safety of permanent BMS implantation for infected aneurysms. Dual BMS placement is only feasible in highly selected patients, with residual sac perfusion carrying a persistent rupture risk until complete thrombosis. Second, long-term safety of this technique lacks large-scale validation, and late complications including recurrent infection, stent biofilm formation, pseudoaneurysm recurrence, and delayed rupture cannot be excluded, warranting prolonged antibiotic therapy and serial imaging surveillance. Third, the definite infection source could not be confirmed. Fourth, the decision to withhold antithrombotic therapy relied on individualized risk–benefit assessment without high-level evidence. Fifth, the optimal duration of suppressive antibiotics after endovascular ISAA treatment remains undefined, and our regimen was individualized. Collectively, this endovascular strategy is not suitable for routine clinical application and requires strict patient selection and lifelong surveillance.

## 4 Conclusions

ISAAs are rare but fatal vascular lesions. For patients who decline or cannot tolerate open surgical repair, overlapping dual BMS implantation serves as a feasible potential treatment alternative under strict selection criteria. Long-term safety and efficacy remain to be further verified. Since infected arterial wall cannot be removed via endovascular intervention, prolonged suppressive antibiotics combined with imaging surveillance are required to prevent infection recurrence and aneurysmal dilatation.

## Data Availability

The original contributions presented in the study are included in the article/Supplementary Material, further inquiries can be directed to the corresponding author.
